# Extracellular Cysteine Proteases of Key Intestinal Protozoan Pathogens—Factors Linked to Virulence and Pathogenicity

**DOI:** 10.3390/ijms241612850

**Published:** 2023-08-16

**Authors:** Raúl Argüello-García, Julio César Carrero, M. Guadalupe Ortega-Pierres

**Affiliations:** 1 Departamento de Genética y Biología Molecular, Centro de Investigación y de Estudios Avanzados del Instituto Politécnico Nacional, México City 07360, Mexico; rag@cinvestav.mx; 2 Departamento de Inmunología, Instituto de Investigaciones Biomédicas, Universidad Nacional Autónoma de México (UNAM), México City 04510, Mexico

**Keywords:** extracellular proteases, cysteine proteases, papain-like proteases, intestinal protozoa, protists, virulence, pathogenicity

## Abstract

Intestinal diseases caused by protistan parasites of the genera *Giardia* (giardiasis), *Entamoeba* (amoebiasis), *Cryptosporidium* (cryptosporidiosis) and *Blastocystis* (blastocystosis) represent a major burden in human and animal populations worldwide due to the severity of diarrhea and/or inflammation in susceptible hosts. These pathogens interact with epithelial cells, promoting increased paracellular permeability and enterocyte cell death (mainly apoptosis), which precede physiological and immunological disorders. Some cell-surface-anchored and molecules secreted from these parasites function as virulence markers, of which peptide hydrolases, particularly cysteine proteases (CPs), are abundant and have versatile lytic activities. Upon secretion, CPs can affect host tissues and immune responses beyond the site of parasite colonization, thereby increasing the pathogens’ virulence. The four intestinal protists considered here are known to secrete predominantly clan A (C1- and C2-type) CPs, some of which have been characterized. CPs of *Giardia duodenalis* (e.g., Giardipain-1) and *Entamoeba histolytica* (EhCPs 1-6 and EhCP112) degrade mucin and villin, cause damage to intercellular junction proteins, induce apoptosis in epithelial cells and degrade immunoglobulins, cytokines and defensins. In *Cryptosporidium*, five Cryptopains are encoded in its genome, but only Cryptopains 4 and 5 are likely secreted. In *Blastocystis* sp., a legumain-activated CP, called Blastopain-1, and legumain itself have been detected in the extracellular medium, and the former has similar adverse effects on epithelial integrity and enterocyte survival. Due to their different functions, these enzymes could represent novel drug targets. Indeed, some promising results with CP inhibitors, such as vinyl sulfones (K11777 and WRR605), the garlic derivative, allicin, and purified amoebic CPs have been obtained in experimental models, suggesting that these enzymes might be useful drug targets.

## 1. Introduction

Protistan parasites of the genera *Giardia*, *Entamoeba*, *Cryptosporidium* and *Blastocystis* cause intestinal infections and diseases (giardiasis, amoebiasis, cryptosporidiosis and blastocystosis) in humans and animals worldwide. Current evidence indicates that these pathogens collectively affect >1.5 billion people and cause ~300,000 deaths per annum [1,2,3]. These figures are likely to get worse due to climate change, extensive human travel and increased globalization. 

All of these parasites colonize the intestinal tract and can cause acute or chronic diarrhea, malabsorption and/or bleeding, with severe sequelae, such as chronic bowel inflammation, reduced growth and development and in severe cases, death. However, infections can be self-limiting or asymptomatic, reflecting a degree of adaptation of these pathogens to their hosts and/or the effectiveness of host immune responses to suppress infections [4]. These pathogens have distinctive life cycles, which are linked to their particular tropism. For instance, *Blastocystis* and *Giardia* are extracellular parasites and *Cryptosporidium* is intracellular/extra cytoplasmic, whereas *E. histolytica* can invade tissues from the large intestine to spread (via the bloodstream) to the liver, spleen, kidney, brain and other organs, enabled by cellular structures for locomotion, attachment and penetration as well as by molecules acting on intra-, epi- or extracellular levels to promote parasite establishment and survival within the host.

Parasite structures and/or molecules that have an adverse effect on host intestinal homeostasis through mechanical, metabolic and functional damage can be virulence factors. These can include surface-anchored or secreted molecules such as toxins, lectins, cysteine-rich proteins, lytic enzymes (proteases, lipases and nucleases), metabolic enzymes and molecules with multiple functions, which are often called “moonlighting” proteins [5,6,7,8]. In the intestinal lumen and in intraepithelial compartments, intestinal protozoa and the molecular products that they release must overcome multiple barriers, such as mucus, epithelial cell surfaces (e.g., via microvilli, receptors and/or channels), intercellular junctions (tight and/or adherent junctions and desmosomes) and elements of the innate and adaptive immune systems (phagocytic/cytotoxic cells, IgA, antimicrobial peptides and cytokines). In this context, peptide hydrolases (proteases) play a key role. Seven broad groups of hydrolytic (i.e., aspartic, asparagine, cysteine, glutamic, metallo-, serine and threonine) proteases are classified in the current release (12.4) of the MEROPS database (https://www.ebi.ac.uk/merops/cgi-bin/family_index?type=P; accessed date: 2 August 2023) [9]. Cysteine proteases (CPs) are the most diverse type (families C1–C124), and tens to hundreds of CPs are encoded in the genomes of intestinal protists. 

Several CPs are synthesized as inactive zymogens, which, upon proteolytic removal of an N-terminal prodomain, become catalytically competent. The catalytic mechanism of CPs involves the following steps: (a) deprotonation of a cysteine at the active site by an adjacent proton acceptor (usually histidine) that renders a thiolate-imidazolium charge, which is stabilized by a neighbor asparagine; (b) nucleophilic attack at the carbonyl carbon by the cysteine thiolate (proton acceptor), which promotes the release of the first half of the product and a thioester intermediate; (c) hydrolysis of the acyl-enzyme intermediate by activated water, which, in turn, releases the second half of the product with a terminal carboxylic acid, converting a CP into its initial state [10]. According to the MEROPS protease database, there are 14 CP groups called “clans” including nine C-type and five P-type groups. Evidence suggests that CPs of intestinal protozoa emerged from convergent evolution of the catalytic mechanism characteristic of clan CA (papain-like); in this clan, the most studied, abundant and relevant CPs are those in families C1 (cathepsin B/L-like) and C2 (calpain-like) [11,12].

In previous reviews, the importance of cysteine proteases in the pathogenesis of some intestinal (*Entamoeba* and *Cryptosporidium*) and extraintestinal (*Trypanosoma*, *Leishmania*, *Plasmodium* and *Toxoplasma*) protozoa has been addressed [11,12]. In this article, we update the knowledge of the nature and roles of CPs of intestinal protozoa (*Giardia*, *Entamoeba*, *Cryptosporidium* and *Blastocystis*) from structural and repertoire-based analyses and discuss the implications of these enzymes in virulence, pathogenicity and modulating host immune responses. Although many articles have reviewed CPs of *Giardia* and *Entamoeba*, reviews of the role of CPs of *Cryptosporidium* and *Blastocystis* are rather sparse. In addition, we focus here particularly on the role of extracellular (secreted) CPs as virulence markers due to their relevance in parasite attachment and invasion of epithelial cells, induction of intestinal cell death and modulation of mucosal responses. We also provide a nomenclature for important CPs of these microorganisms and suggest avenues for further discovery work.

## 2. Cysteine Proteases Secreted by *G. duodenalis*: An Arsenal of Cathepsin-like Enzymes with Multiple Targets

*Giardia duodenalis* (syn. *G. lamblia*, *G. intestinalis*) is an intestinal protozoan parasite that causes giardiasis in humans and livestock, companion and wild animals. Giardiasis is a leading cause of waterborne diarrheal infections worldwide, with an estimated number of 280 million human cases per annum. Giardiasis is included in the WHO’s Neglected Disease Initiative [13], and *Giardia* is considered a potential bioterrorism agent (category C) [14]. During its life cycle, *Giardia* alternates between the vegetative, pathogenic trophozoite and dormant, infective cyst forms. When a susceptible host ingests cysts contaminating water and food, excystation occurs during passage through the stomach into the small intestine. Emerging excyzoites rapidly divide by binary fission to give rise to trophozoites, which adhere to enterocytes via their adhesive disks and proliferate and progressively colonize the epithelial surfaces of the duodenum and midjejunum. Here, trophozoites are exposed to an alkaline pH and biliary secretions; cholesterol deprivation induces encystation, leading to the formation of cysts, which are shed in the feces. 

*Giardia* infection can result in disease (giardiasis), which is symptomatic (acute to chronic) but can also be asymptomatic. The pathogenesis of giardiasis relates to virulence factors, which become active at the interface between trophozoites and small intestinal epithelium and are produced/released by trophozoites. These factors include cathepsin B proteases, tenascins, cystatin and a number of variant-specific surface proteins (VSPs) [15]. Of the secreted products, CP activity accounts for almost all of the parasite-derived proteolysis from supernatants recovered from trophozoite–epithelial cell interaction assays [16]. 

It has been reported that the genome of *G. duodenalis* (reference clone WBA6) encodes 25 CPs. Of these, 21 are cathepsin type: 8 cathepsin-L, 9 cathepsin-B with endopeptidase activity, 3 cathepsin-B with endopeptidase and dipeptidyl peptidase activity (associated with the presence of an ‘occluding loop’) and 1 cathepsin-C-like (the encystation-specific CP, ESCP) [17]. The repertoire of the cathepsin-like gene varies among strains and assemblages. Thus, in assemblage B, the GS strain has 17 homologs, while strain GS-B has 23; in assemblage E, the P15 strain contains 23 homologs. These proteases are activated following proteolytic removal of the N-terminal prodomain of their inactive precursors. During trophozoite growth and encystation, differential expression of cathepsin-B-like proteases occurs, including those formerly named CP2, CP4 and CP5, as well as the cathepsin-L-like protease CP17 [18], which are abundantly expressed [19]. This finding suggests that this family of proteases has diverse functional roles, depending on their location in the cellular compartments. These proteases, which are expressed and released by the parasite, have gelatinolytic and/or collagenolytic activities [16,20].

**Figure 1:**
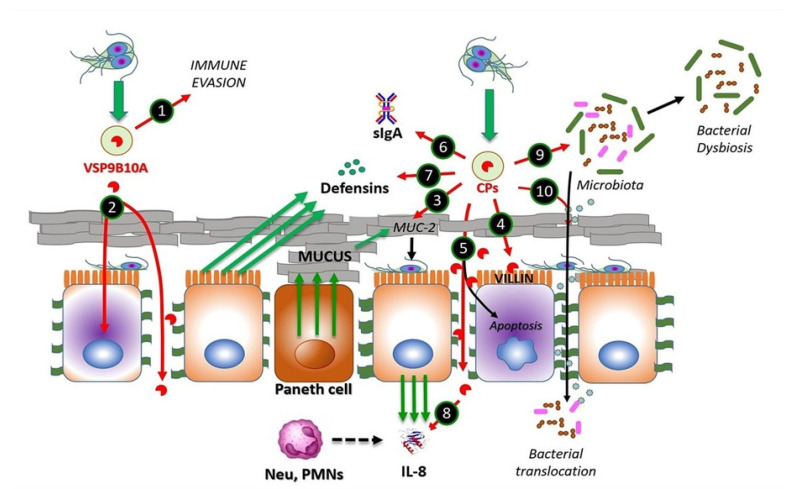
CPs from *Giardia duodenalis* trophozoites interact with multiple targets in the small intestine. Experimental models of trophozoites–epithelial cell interactions indicate that the parasite releases CPs, mainly cathepsin B-type, including Giardipain-1 (GL50803_14019), GL16160 and GL16779 and non-canonical CPs (e.g., VSP9B10A). This latter CP may serve, when expressed, to divert the immune system (1) or may cause damage to the epithelium due to a loss of cell–cell junction and cytotoxicity in the cell (2). Secreted CPs have been shown to degrade substrates, such as mucin 2, an important component of intestinal mucus (3), enabling trophozoite adhesion to microvilli. Direct damage to epithelial cell integrity by CPs may include disruption of the cytoskeletal microvillus-resident protein villin (4) and the disruption of tight junction proteins, such as ZO-1 and claudins, also involving adherens junction proteins including β-catenin or E-cadherin (5). Soluble elements of the innate immune response, including secretory IgA (6), produced by plasmatic B-cells, defensins (7) and IL-8 (8), both produced by epithelial cells, of which the latter works as neutrophil attractant, might be degraded by giardial CPs. These enzymes also provoke alterations in the microbiome of the small intestine, leading to dysbiosis (9), while bacterial translocation from the luminal to the intraepithelial compartment (10) may be promoted via the degradation of intercellular junctions by CPs as mentioned. Recent studies suggest that giardial CPs might be secreted after removal of N-terminal prodomain and inclusion into membrane-bound extracellular vesicles (EVs), mainly exosomes (green-filled circles).

Interestingly, in *G. duodenalis*, some CPs are also encoded by other genes representing >230 variant surface proteins (VSPs). These VSPs are expressed as glycoproteins and/or palmitoyl-proteins, and their genes are transcriptionally regulated by an iRNA-silencing mechanism (named “all but one”) and are also involved in antigenic variation and the induction of protective immune responses. In this family, there is at least one clade of CPs with structural and/or functional similarities to cathepsins B. One of them is VSP9B10A (739 amino acids (aa) long; GL50803_101074), which can be detected in a secreted form (~75 kDa) during trophozoite–epithelial cell monolayer (IEC-6) interactions and displays proteolytic activity [21]. VSP9B10A contains a central region (aa 324 to 684) with a catalytic substrate binding pocket and calcium recognition sites of a typical cathepsin B. This atypical cathepsin is not always expressed on the trophozoite surface but has been shown to be toxic to epithelial monolayers [21], leading to a loss of cell-cell and cell-substrate contacts (Figure 1). Experimental evidence has demonstrated that VSP9B10A is a conditional virulence factor [21]. 

*Giardia* CPs might be regulated at the intracellular level by cystatins, as is the case for *Cryptosporidium* (cryptostatin) [22]. Cystatins comprise a superfamily of proteins with a homologous sequence and structure, consisting of an antiparallel β-sheet topped with an α-helix. These function as endogenous cysteine protease inhibitors of target proteases, such as cathepsins B, C, L and H, and are currently classified into type 1 (stefins), type 2 (cystatins), type 3 (kininogens), latexins and fetuins [23]. In *G. duodenalis*, a stefin (GL50803_27918) was identified as a cytoplasmic protein that potently inhibits three major secreted CPs of the clan CA papain-type, namely GL50803_14019 (Giardipain-1), _16160 and _16779. Interestingly, stefin is a weak inhibitor of human cathepsin B [24] and might play a role in *Giardia*–epithelial cell interaction, as previous evidence revealed an upregulation of cathepsin-L-like proteins GL50803_137680 or GL_50803_3099 [25] upon the interaction of distinct strains (WB, P-1, GS/M or NF) of *Giardia* with ICE-6 monolayers [26]. Another interaction study [27] revealed conspicuous expression of cathepsin B-like proteases in the WB strain of *Giardia* (i.e., GL50803_16160, _16468 and _16779) and their homologs in the GS strain (i.e., GL50581_2946, _438 and _78), in addition to other cathepsins B (including GL50803_14019 and GL50581_2036). A proteomic study of the same strains (using the same intestinal cell line) reported a distinct repertoire of three cathepsin B-like proteases in the WB strain of *Giardia* (i.e., GL50803_15564, _16468 and _17516) and their closest homolog in the GS strain (GL50581_2036, _438 and _2318) [28]. This information suggests that trophozoites of the zoonotic assemblages A and B (represented by the strains WB and GS, respectively) exhibit similar CP secretion profiles upon interaction with epithelial cells. 

Following secretion, these proteases become catalytically competent due to the absence of an occluding loop and the intracellular excision of prodomains. In the case of Giardipain-1 (GL50803_ 14019, formerly named CP2), its precursor (33 kDa) is 8 kDa larger, i.e., 76 amino acids (aa) longer than the mature protein (25 kDa), which possesses gelatinolytic activity and exhibits pro-apoptotic effects on IEC-6 and epithelial cell (MDCK) monolayers [29]. The CPs secreted by *Giardia* have different protein targets on host epithelial cells and eventually disrupt intestinal homeostasis, in turn causing small intestinal damage [30] (cf. Figure 1).

The mechanism underlying the secretion of *Giardia* CPs is not yet fully understood. However, there is evidence for the processing of the N-terminal prodomain of clan CA proteases, such as Giardipain-1 [18,29]. In a recent proteomic study of extracellular vesicles (EVs; <100 nm in size) released by *Giardia* trophozoites, cathepsin B-like proteases have been identified as cargo within exosomes [31] (Figure 1). Considering the role of EVs in the intercellular crosstalk between parasites and intestinal epithelial cells, it is likely that these vesicles may mediate the release of cargo proteins around or within epithelial cells to promote cellular and tissue damage.

One of the first barriers that trophozoites encounter is the mucus layer on the brush border of the intestinal epithelium. This layer is mainly composed of membrane-bound mucins, such as MUC4 and MUC16, and gel-forming mucins, such as MUC2, the latter of which predominates in the gastrointestinal tract [32]. Interestingly, the use of a human mucus-producing colonic cell line (LS174T) in interaction assays using excretory/secretory proteins (ESPs) from strains NF (assemblage A) and GS/M (assemblage B) revealed a concentration-dependent degradation of MUC2, which was abrogated by pre-treatment with a CP inhibitor, called E-64 [33] (Figure 1). In addition, a recent investigation [34] reported a novel mechanism by which *Giardia* CPs modulate goblet cell activity via the cleavage and activation of protease-activated receptor 2 (PAR-2), which can regulate mucin gene expression in intestinal goblet cells. 

Another barrier against *Giardia* is the brush border at the apical surface of enterocytes. However, it has been shown that trophozoites adhering to the microvillus lining of the enterocytes cause damage to the intestinal epithelium, leading to structural alterations and decreased absorptive surface area [35]. In this context, villin, an actin-binding protein expressed in the microvillus layer of enterocytes, can be degraded and redistributed by the action of surface-exposed *Giardia* CPs (Figure 1), supported by evidence showing the intact villin structure of Caco-2 cells exposed to trophozoites pre-incubated with inhibitors, such as the membrane-permeant E-64 or ML-9 (inhibitor of myosin light chain phosphorylation, MLCK) [36]. 

Further damage by proteases secreted by *G. duodenalis* trophozoites can also occur between cells in the intestinal epithelium. This has been shown via the disruption of transmembrane, plaque and filamentous proteins intervening apical junctional complexes (AJCs), known as tight junctions (claudins, occludins and/or zonula occludens—ZO-proteins), and adherent junctions (E-cadherin and β-catenin) [29,37]. There is clear evidence that *Giardia* ESPs, when added to Caco-2 cells, can alter the distribution of AJC components, such as claudin-1, occludin, ZO-1 and intracellular F-actin [37]. Furthermore, when added to IEC-6 monolayers, purified secreted protease Giardipain-1 has been shown to colocalize with claudin-1 and occludin at cell junctions, affecting distribution, decreasing transepithelial electrical resistance and inducing apoptosis, leading to pathological changes in epithelial cells [29]. Other experiments have shown that recombinant forms of each of the three secreted CPs, designated GL50803_14019 (Giardipain-1), GL50803_16160 and GL50803_16779 (formerly CP3) [27], affect the distribution of AJC proteins [37] and also degrade E-cadherin and occludin, which could be reduced by inhibitor E-64d [37]. Further, the three proteases degraded claudin-1 and -4 and β-catenin, respectively, suggesting that the limited number of CPs released by trophozoites have a redundant activity on cell junction proteins (Figure 1). The proteolytic activity of secreted proteases on specific substrates was shown using recombinant proteins in a two-thioredoxin system. Activity was associated with Giardipain-1, GL50803_16160 and GL50803_16779. Similar substrate selectivities were observed for Giardipain-1 and GL50803_16160; however, these differed from GL50803_16779 [24,38]. 

Bioinformatics analyses performed to screen the human small intestinal proteome to determine the cleavage consensus sequence of GL50803_16779 predicted immunoglobulins (Ig), defensins (Df) and chemokines (Ck) as targets, suggesting that parasite proteases interfere with adaptive host immune responses (Figure 1) [24,37,38,39,40]. Moreover, the different proteases degraded recombinant human defensins αDf1, βDf5 and αDf6, whereas the activity of Giardipain-1 was higher than those recorded for other proteases. Giardipain-1 may participate in neutralizing the innate response induced by Paneth cell defensins [24,38]. Chemokines/cytokines produced by epithelial cells, including CXCL1–3, are upregulated upon exposure to trophozoites [39]. In this context, it has been shown that interleukin-8 (CXCL8) is degraded by *Giardia* CPs, thereby reducing neutrophil recruitment and inflammatory responses [40], while recombinant (and upregulated) chemokines/cytokines CXCL1–3, CXCL8, CCL2 and CCL20 were cleaved by either ESPs or recombinant GL-50803_16160 [37]. These studies suggest that CPs participate in the onset and establishment of *Giardia* infection and also modulate host immune responses.

In experiments in which device-isolated biofilms and microbiota-free mice were used, it was observed that secreted CPs play a role in the interplay of *Giardia* with the commensal microbiota communities located in the gut as biofilm-forming, planktonic bacteria. In these studies, a proinflammatory response was observed, even in the absence of *Giardia*, suggesting that CPs from *Giardia* play a role in intestinal disease, including irritable bowel ensuing infection [41,42]. Interestingly, recent experiments from our group [30] have shown that, in an experimental infection system, purified Giardipain-1 induces apoptosis and the extrusion of epithelial cells at the tips of the villi in ligated intestinal loops in jirds (*Meriones unguiculatus*). Moreover, the infection of jirds with trophozoites expressing Giardipain-1 resulted in intestinal epithelial damage, cellular infiltration, crypt hyperplasia, goblet cell hypertrophy and edema. Pathological alterations were pronounced when jirds were infected (via gavage) with *Giardia* trophozoites that stably overexpressed Giardipain-1. Furthermore, *Giardia* colonization in jirds resulted in chronic inflammation, which might relate to dysbiosis triggered by the parasite (Figure 1). These results demonstrated that Giardipain-1-induced alterations seen in the intestinal epithelial cells and in the microbiome contribute to the pathogenesis of giardiasis and its associated complications [30]. However, experimental evidence indicates that it is possible that other major secreted proteases such as GL50803_16160 and GL50803_16779, which are structurally similar to Giardipain-1, may have similar effects during *G. duodenalis* infection.

## 3. *Entamoeba histolytica* Cysteine Proteases: Repertoire, Pathogenic Role, Regulation and Possible Intervention Targets

*Entamoeba histolytica* causes intestinal amoebiasis in humans, which is a major problem in developing countries worldwide. The species name “histolytica” (assigned by Schaudinn [43]) relates to the tissue damage that this parasite causes in its host. Dobell [44] agreed with this name, stating that this amoeba secretes "a potent cytolytic ferment" that dissolves tissues rather than causing physical destruction via pseudopods. The first study of proteolytic enzymes in axenic cultures of *E. histolytica* was carried out in the late 1970s [45]. In this study, two proteinases were identified from *E. histolytica* solubilized in butanol, an acidic one similar to cathepsin D and a neutral one similar to cathepsin B, and the latter was inhibited by iodoacetamide and activated by cysteine or dithiothreitol. Subsequently, Lushbaugh et al. [46] demonstrated the presence of cytopathogenic activity in cell-free extracts, and they purified, by gel filtration, a cytotoxic substance that damaged cell monolayers and proposed to be a virulence factor associated with the development of ulcers and diarrhea. Later, Bos et al. [47] also observed this activity in the products excreted from live amoeba and suggested that, in addition to the cytopathic effect, this substance could also be responsible for the contact-dependent lysis caused by *E. histolytica*. However, Ravdin et al. [48,49] reported that contact-dependent lysis was not inhibited by serum, as was the cytotoxic substance released into the medium, suggesting that the molecules involved in both events were different, being phospholipase C and the amoebapore, two candidates linked to cytotoxic effect(s). The cytotoxin was purified by McGowan et al [50], who demonstrated that it did not have hemolysin, gelatinase, lipase, glucosaminidase or catalase enzymatic activities but did have cytotoxic activity that could be reversed with protease inhibitors, suggesting that it was a different type of proteinase. The activity of this cytotoxin was increased by thiol-reducing agents, such as dithiothreitol (as previously reported), and it was preferentially detected in the secretions of saline-incubated amoeba [51]. In the same study, a correlation was found between the level of activity of these proteolytic enzymes and the ability to detach tissue culture cells from their substrate. 

In these studies, it was suggested that the amoeba’s ability to invade tissues was linked to its virulence. In this regard, Lushbaugh et al. [52] demonstrated that the more virulent strain HM-1 secreted more cytotoxin in the presence of tissue extract than the less virulent strain KH-9 and that this activity had characteristics of a neutral cathepsin-like proteinase B (cysteine proteinase) which was inhibited by non-immune serum or by anti-amoeba IgG antibodies. However, its inactivity on synthetic substrates with a single amino acid residue for cathepsin and its activity on natural substrates suggested that this could be cathepsin L. Finally, cathepsin B from *E. histolytica* was purified by ion-exchange chromatography and was shown to have proteolytic activity on hemoglobin, azocasein and the specific substrate of the enzyme carbobenzoxy-L-arginyl-7amino-4-trifluoromethylcoumarin (Z-arg-arg-AFC), the latter of which was inhibited by p-chloromercurybenzoate and reversed by free sulfhydryl groups typical for cysteine proteinases [53]. The collagenolytic activity of the amoeba cytotoxin was also suggested to be related to cathepsin B [54]. Due to its wide range of actions in terms of pH and variety of substrates, cathepsin B is considered the main digestive and/or lysosomal enzyme of *E. histolytica* (see [55]). Other proteinases identified in this species include cathepsins D, G and L, and these have a minor role in the secreted proteolytic activity of the amoeba.

The main cathepsins of *E. histolytica* identified have a cathepsin-L-like structure fold but similar substrate specificities to cathepsin-B [56,57]. These proteases are inhibited by the inhibitor l-trans-epoxysuccinyl-leucylamido-(4-guanidino) butane (E-64) but not by the serine protease inhibitor phenylmethylsulfonyl fluoride. Amoebic cysteine proteins are also inhibited by sulfhydryl reagents (p-chloromercuribenzoate [PCMB]) and activated by dithiothreitol and 2-mercaptoethanol. Although at least 50 CP genes have been identified in the *E. histolytica* genome, no more than 6 genes seem to be expressed under axenic culture conditions [56,58]. The analysis of *E. histolytica* extracts has shown several proteinases in the range of 16 kDa to ~150 kDa [59,60], but, in particular, four to six bands of proteinase activity are usually identified on gelatin-polyacrylamide gels. The first similar, but distinct, cysteine proteinase activities purified and characterized from *E. histolytica* were amebapain [61] and histolysin [62], now known as EhCP1 and EhCP2, respectively. Together with EhCP5, which was identified as the main surface-localized CP of the parasite [63], these three proteases were found to be highly expressed in trophozoites of *E. histolytica* to such an extent that they represent 90% of CP-encoding transcripts and virtually all of the CP activity identified in the amoeba [64]. In the same study, three other CP genes encoding EhCP3, EhCP4 and EhCP6 with limited transcription were identified. Together with those encoding EhCP1, EhCP2 and EhCP5, the genes are 40% to 85% different in nucleotide sequence [64].

Interestingly, considerable amounts of CPs were found in the culture supernatants of intact *E. histolytica* trophozoites, suggesting that they are actively secreted into the extracellular milieu, which appears not to be the case for the amoeba pore-forming peptide [65]. Except for EhCP1 and EhCP5, homologs of the other cysteine proteases were found to be expressed in the nonpathogenic human amoeba *Entamoeba dispar* (see [64]), widely used as a reference for identifying pathogenicity-associated molecules. Therefore, this result suggested that EhCP1 and EhCP5 could be responsible for most of the pathogenic potential of *E. histolytica*. Noteworthily, the overexpression of the gene encoding EhCP5, in addition to increasing its proteolytic activity, also increased the activity of EhCP1 and EhCP2 [66]. This information suggests an enzyme-converting activity for EhCP5 and explains the high contribution to the proteolytic activity of *E. histolytica* trophozoites. Another CP was described by García-Rivera et al. [67], who detected it on the trophozoite surface associated with an adhesin forming a 112 kDa complex (EhCP112). In general, the subcellular location of amoeba CPs is highly variable, and they have been detected by immunohistochemical methods in amorphous areas of the cytoplasm. These areas could be lysosome equivalents, and they can be translocated to phagocytic vacuoles when erythrocytes are ingested or moved to the surface, to be secreted [68]. Another study suggested that CPs of *E. histolytica* reside in compartments that communicate with, or are part of, the endosomal pathway because early and late endosomes purified from trophozoites showed intense CP activity that was associated with the presence of small Ras GTPases [69]. Co-immunoprecipitation studies using EhCP5 allowed the identification of a new family of receptors that mediate the trafficking of lysosomal hydrolases in the amoeba. Of a total of 11 members identified, the 110 kDa CP-binding protein family 1, CPBF1, was found to be the only one responsible for binding to the main CPs of *E. histolytica* and for mediating EhCP5 trafficking from the endoplasmic reticulum to lysosomes, regulating the intracellular transport of CPs in the amoeba [70,71]. Other types of proteases, such as serine proteases, have also been identified in *E. histolytica*, but they seem to contribute much less to the proteolytic activity detected in this parasite [72]. General characteristics of the main seven *E. histolytica* CPs are shown in Table 1.

CPs are well studied in *E. histolytica* and are recognized as amoebic virulence factors linked to invasion and tissue damage in the host. *E. dispar*, the nonpathogenic/non-invasive sister amoeba of *E. histolytica*, shows 10- to 1000-fold less CP activity in its extracts compared with *E. histolytica*, which is associated with the absence of expression in *E. dispar* of the gene that codes for the largest CP of the amoeba, EhCP5, due to its high loss throughout evolution [91,92]. The same observation has been made with low or non-virulent *E. histolytica* isolates and clones, which have shown little or no CP activity or limited cytopathic effects [93]. This contrasts highly virulent clones of *E. histolytica* derived from passages in the liver of hamsters, such as clone 1659, which exhibit very high enterotoxicity associated with an increase of two CPs of 35 and 75 kDa in its secreted products [94]. 

In vitro and in vivo studies also suggest that the activity of secreted amoebic CPs is essential for the invasive process. They are responsible for the cytopathic effect of supernatants and extracts of the parasite that leads to the detachment of the cells in the monolayers, since specific inhibitors of CPs completely prevent this effect [91]. Among the extracellular matrix molecules that have been identified as targets of these proteases are collagen, fibrinogen, laminin and elastin, and the breakdown of these molecules has been associated with the ability of the parasite to lead to invasive amoebiasis [62,95,96]. Recombinant EhCP5 has been found to be capable of degrading collagen, fibrinogen, hemoglobin, albumin, gelatin, mucin and IgG [97]. The degradation of the extracellular matrix by CPs in epithelial cell monolayers leads to cell detachment, involving anoikis, a type of apoptotic cell death dependent on JNK phosphatase activity that occurs independent of contact [98]. 

**Figure 2:**
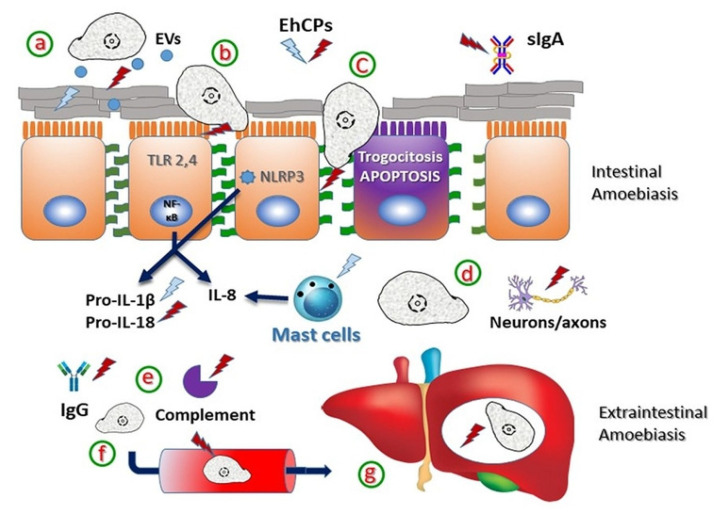
*Entamoeba histolytica* CPs (EhCPs) are central to invasion, pathogenicity and immune evasion during intestinal and extraintestinal amoebiasis. Once the amoebic trophozoite reaches the mucus layer in the large intestine (**a**), EhCPs released in extracellular vesicles (EVs) can degrade the mucin backbone and secretory IgA antibodies (sIgA), which facilitates initiation of mucosal invasion (degradation is indicated by red rays). Intriguingly, the participation of EhCPs in the secretagogue capacity of the amoeba has also been described (activation is indicated by the blue rays). During trophozoite contact with the apical region of the intestinal epithelium (**b**), EhCPs degrade components of the extracellular matrix and villin in the apical region of enterocytes, eroding the epithelium and activating signaling pathways that lead to nuclear translocation of the transcription factor NF-κB, the inflammasome assembly and the expression of proinflammatory cytokines. The penetration of the amoeba through the epithelium (**c**) occurs in the intercellular spaces by EhCPs degradation of tight junctions, adhesion junctions and desmosomes components. At the same time, the parasite induces the death of enterocytes by trogocytosis and apoptosis, with EhCPs participating in the former. Since the amoeba is in the submucosa (**d**), the EhCPs can activate mast cells to produce IL-8, while they can degrade cytokines such as Pro-IL-18, or in contrast, activate them as in the case of Pro-IL-1B. At this point, EhCPs can also destroy intestinal nervous tissue (neurons/axons), affecting its physiology. During the invasion of the tissue, the amoeba also comes in contact with blood components (**e**), such as complement and IgG antibodies, which are degraded by EhCPs. At this point, in very sporadic cases and for reasons not yet understood, amoebae can migrate through the portal vein to the liver (**f**), where EhCPsbreak down hemoglobin to use iron. Finally, once in the liver (**g**), EhCPs contribute to tissue damage through the degradation of cell matrix components, enterocytes, and recruited immune cells, leading to the development of amoebic liver abscesses.

In the context of intestinal invasion, the amoebic trophozoite must be able to overcome the barrier of mucus embedded with IgA antibodies and antimicrobial peptides. Studies using high molecular weight LS 174T cell mucin metabolically labeled with 35S cysteine showed that amoeba CPs degraded it in a time- and dose-dependent manner, generating lower molecular weight fragments that were 38% less effective in inhibiting the adhesion of trophozoites to monolayers of CHO epithelial cells [99]. The same group showed that a large part of this activity was due to EhCP5, since trophozoites of *E. histolytica* deficient in the production of this CP by using an antisense transcript showed more than a 60% decrease in their ability to degrade the mucin, while recombinant EhCP5 alone degraded it by more than 45% [83]. Ironically, the amoeba has a potent secretagogue activity on goblet cells of the colon, a process that is also mediated by CPs and particularly by EhCP5 through its binding to the αvβ3 integrin and a PI3K, PKCδ and MARCKS-signaling-dependent pathway [85]. Embedded in the mucus, amoeba can encounter secretory IgA antibodies and complement components, which are degraded by CPs, and it is described in more detail below (section on interference with the immune response). Once the amoeba bypasses the mucus and reaches the surface of the intestinal epithelium, the CPs degrade the actin-bundling protein villin on the apical surface of the enterocytes, as determined by Western blot assays on Caco-2 cells [100]. In parallel, EhCP112 dissociates from the amoebic surface, penetrates through the intercellular spaces and interacts with claudins 1 and 2 at tight junctions and with β-cat, E-cad, Dsp l/ll and Dsg-2 in adhesion junctions and desmosomes, to which it delocalizes and degrades, favoring intercellular separation and allowing the invasion of the epithelium [89,90]. As a result of tissue invasion, the amoeba comes into contact with blood; CPs, including EhCP112, bind to red blood cells and degrade hemoglobin to obtain iron, an essential component for their growth [88,101]. Likewise, the digestion of erythrocytes once phagocytosed also depends on the CPs, since trophozoites silenced for the expression of the major peptidases (EhCP1, 2, 5 and 7) showed a marked reduction in their hemolytic capacity but not in their phagocytic capacity [76]. Studies using the specific membrane-permeant inhibitor of CPs, E-64d, confirmed that the activity of these parasite proteases is not required for phagocytosis but that they are necessary for amoeba-mediated trogocytosis, a type of cell death caused by ingestion of fragments (bites) from living cells [102]. However, a knockdown study on the expression of EhCP112 in trophozoites using short-hairpin RNAs showed not only deficiency in various virulence-related functions such as adhesion and cytotoxic and cytopathic effects on MDCK cell monolayers but also in erythrophagocytosis [103]. In addition to epithelial cells and mucin, the destruction of neurons and axons has been reported in a culture model by the proteolytic action of soluble extracts or secreted components of the amoeba that are inhibited by the E-64d protease inhibitor. The results suggested that amoebic CPs are responsible for the reduction of >75% of axons in the murine model of cecal amoebiasis ([104]; Figure 2).

Once *E. histolytica* has overcome the physical and chemical barriers at the intestinal milieu, it spreads via the portal vein to other tissues, mainly the liver, where it usually leads to the development of liver abscesses. Study of extraintestinal amoebasis in experimental models has shown that the activity of parasitic CPs is important for tissue pathology, especially for the cytolytic but not cytotoxic effect on liver cells [105]. Thus, for example, treatment of virulent *E. histolytica* trophozoites with the membrane-permeant specific inhibitor of CPs, E-64d, prevented the formation of liver abscesses in mice with severe combined immunodeficiency (SCID) and in golden hamsters (*Mesocricetus auratus*) [106,107]. The passage of axenic or xenic *E. histolytica* trophozoites through the liver of golden hamsters resulted in a progressive increase in proteinase activity of amoeba supernatants and lysates, which correlated well with increased virulence [108]. However, the degree of relevance of each CP in the development of liver abscesses seems to be variable. Thus, although trophozoites of both *E. histolytica* and *E. dispar* transfected with an episomal vector that drives the overexpression of EhCP2 showed a greater ability to kill monolayers of cells in vitro, they did not show changes in their ability to induce amoebic liver abscesses [97]. Unfortunately, the participation of the other main CPs of the amoeba in culture, EhCP1 and EhCP5, could not be evaluated as no transfectants were found to overexpress them. According to the above, EhCP2 purified from *E. histolytica* HM1: IMSS trophozoite extracts and encapsulated in inert resin microspheres, when injected into the hamster portal vein, caused only slight inflammation in the liver tissue. This observation, in combination with the fact that EhCP2 was not found outside of the amoeba in liver abscess tissue sections, suggest that this protease plays little or no role in the pathology of experimental hepatic amoebiasis [109]. Interestingly, the expression of CPs appears to change dramatically when analyzed in tissues. Unlike what has been repeatedly reported in axenic cultures, where more than 90% of the activity is due to EhCPs1, 2, 5 and 7, a study aiming to evaluate the expression of the CP genes of *E. histolytica* during the formation of amoebic liver abscesses by real-time PCR, found that other EhCPs increase their expression depending on the experimental model: Ehcp-a3, -a4, -a10 and -c13 during infection in gerbils and mice and Ehcp -b8 and -b9 only in gerbils [110]. Of these, the participation of CPs b8, b9 and c13 in the pathology was demonstrated by restoring the capacity of the non-virulent *E. histolytica* clone A1 to develop amoebic liver abscesses [110]. Likewise, the critical participation of EhCP5 in the development of abscesses was shown by the fact that trophozoites transfected with an antisense probe for this gene, in addition to having only 10% of the CP activity, lost the ability to develop abscesses in hamsters [111]. Similar results were observed with trophozoites deficient in the expression of EhCP112 using a short-hairpin RNA, which reduced their ability to induce amoebic liver abscesses in hamsters by more than 60% ([103]; Figure 2).

The secretion of CPs by *E. histolytica* trophozoites seems to be a highly regulated process, in which external stimuli are of significant importance. One of these stimuli is type 2 collagen and Ca^2+^, which led to a two-fold increase in the secretion of CPs of the HM1: IMSS strain, an increase that was inhibited by cytochalasin D, suggesting the participation of the parasite cytoskeleton in this process [112]. Iron is also an external factor that modulates the expression of amoeba virulence factors, including CPs. Thus, *E. histolytica* possesses an iron stress response that leads to increased expression levels of the EhCPs1-6 genes under iron-restricted conditions [86]. The co-incubation of *E. histolytica* trophozoites with *Escherichia coli* increases the expression of the EhCP2 and EhCP5 genes while enhancing the activity of the CPs by 3–6-fold, which resulted in greater cytotoxicity on BHK cells [113]. On the other hand, the amoeba has internal regulators of the expression of CPs, such as cyclooxygenase (EhCox), since silencing this gene led to a strong increase in the endogenous expression of CPs, which correlated with an increase in the ability of these amoebas to phagocytose, to damage colonic epithelial cell cultures and to induce proinflammatory cytokines and cell infiltrates in intestinal loops of mice [114]. Intracellularly, amoeba CPs have been identified in abundance in early and late endosomes colocalizing with small GTPases of the Ras superfamily, such as EhRab7A and EhRab11A [69]. Indeed, there is clear evidence that the trafficking and secretion of amoebic CPs are regulated by these Rabs. During phagocytosis, trafficking appears to be regulated by EhRab5 and EhRab7A [115], while EhRab11B has been associated with secretion [116]. Thus, once EhRab11B was overexpressed in the amoeba, the secretion of CPs into the medium as detected by immunoblot assays increased 18, 4.2 and 2 times for EhCPs 1, 2 and 5, respectively, while in the trophozoite extract, it increased 6.6, 3.2 and 2 times, respectively; the increased expression was reflected in a greater ability to damage CHO cell monolayers [116]. These results suggest that the release of CPs from the amoeba occurs through processes of vesicular trafficking and secretion regulated by their small GTPases.

The ability of *E. histolytica* to evade the immune response is widely recognized. This effect is mainly due to the proteolytic activity of CPs. In the late 1980s and early 1990s, it was reported that the 56-kDa amoeba-neutral CP secreted into the extracellular medium could cleave the C3 component of complement in the fluid phase to C3b or C3bi, which may lyse the nonpathogenic amoeba, but not the pathogenic one [117]. Although it was suggested that the activation of the alternative complement pathway by this C3 disruption may play a role in the early inflammatory response in amebic lesions contributing to the pathogenesis of invasive amoebiasis, the same group subsequently demonstrated that CPs may also degrade the proinflammatory C3a and C5a anaphylatoxins into biologically inactive fragments, thus avoiding the host’s immune response [118]. Likewise, the neutral CP of *E. histolytica* also rapidly degraded human IgA immunoglobulin, both in serum and in secretions, suggesting that it could be important during the process of intestinal invasion by amoeba [119]. Furthermore, both secretory IgA1 and IgA2, as well as their secretory component, were also degraded by a CP activity located on the surface of glutaraldehyde-fixed amoebae, which could be EhCP5 [63,120]. The degradation of human polyclonal IgG and murine monoclonal IgG has also been documented [121], suggesting that CPs play an important role in the evasion of the immune response by limiting the effectiveness of the humoral response.

Cellular response mediators are also targeted by amoebic CPs. It has been shown that CPs can mimic the action of caspase-1, processing the IL-1beta precursor (Pro-IL-1beta) and generating active IL-beta, thereby contributing to inflammation and tissue damage in a model of human intestinal xenografts in SCID mice [74]. This correlates with results showing the ability of EhCP5 to stimulate the proinflammatory response mediated by the transcription factor NF-kB in Caco-2 colonic cells through its binding to the α(v)β (3) integrin leading to activation of a downstream signaling cascade resulting in Akt-473 phosphorylation and NEMO ubiquitination [122]. However, in the case of Pro-IL-18, both the proteinases from an amoebic lysate and the recombinant EhCP5 expressed in yeast, instead of activating the precursor, cleaved it into small biologically inactive peptides [77]. However, a study using the secretion products of *E. histolytica* trophozoites suggests that the CPs present in the supernatant of activated human mast cells (HMC-1) express and produce IL-8 via a protease-activated-dependent mechanism receptor 2 (PAR2), a process that was inhibited by the E-64 inhibitor [123]. Unfortunately, the activity of IL-18 released by the cells was not evaluated, so the effect of CPs on this cytokine once produced is not known.

Regarding chemokines, it has been observed that cellular immunity molecules responsible for the chemotaxis of leukocytes to the site of infection, such as CCL2, CCL13 and CXCL8, are rapidly degraded by EhCP2, generating very low molecular weight fragments that show decreased ability to attract THP-1 cells, when compared to untreated chemokines [78]. However, in the case of a CXCL8 variant that has five more amino-terminal residues, treatment with EhCP2 increased its chemotactic activity, generating in this case a product that was more active [78]. This suggests that although amoeba CPs tend to cleave cytokines and chemokines into biologically inactive fragments, in some cases, they can generate more active derivatives, implying that they can interfere with or modulate leukocyte chemotaxis, thus circumventing the host’s immune response.

Due to their critical role in pathogenesis, EhCPs, such as those of other parasitic protozoans, may be druggable targets based on results obtained using in vitro and in vivo models. In this regard, several inhibitors have been developed. A specific vinyl sulfone inhibitor, WRR605, the synthesis of which was based on the substrate specificity of EhCP4, inhibited the recombinant enzyme in vitro and significantly reduced parasite burden and inflammation in the mouse cecal model [81].

Amoebic CPs are potent immunogens, as they induce high levels of antibodies in infected humans and animals. Thus, up to 84% of human salivary samples from patients with intestinal amoebiasis recognized a purified 70 kDa CP on Western blot, and the specific secretory IgA antibodies inhibited the proteolytic activity of *E. histolytica* extracts [124]. Based on this finding, CPs have been proposed as targets for the development of new treatments and vaccines. In this context, a protein band with CP activity of around 56–66 kDa present in the supernatant of *E. histolytica* trophozoite cultures has been used in vaccination trials in animal models. The immunization of golden hamsters with this band resulted in 62.5% protection against the development of amoebic liver abscesses and the generation of high levels of antibodies against the protease; however, no correlation was observed between antibody titers and protection [125]. Another study evaluated the potential of an *E. histolytica* surface metalloprotease called EhMSP-1, homologous to the *Leishmania* GP63 protein, as a vaccine antigen. This protease was recognized by 75% of the sera of African patients (n = 12) with intestinal amoebiasis [126]. The immunization of golden hamsters intraperitoneally with four fragments of EhMSP-1 resulted in a combined protection of 68% against the development of liver abscesses, which correlated with the generation of specific IgG antibodies with lytic capacity against amoebic trophozoites in the presence of a complement [126]. These studies suggest that these proteases could be vaccine candidates. 

## 4. Cysteine Proteases of *Cryptosporidium*: A Family Affair and Beyond

*Cryptosporidium* is a species complex of apicomplexan alveolate protists, which cause intestinal infections, namely cryptosporidiosis. In humans, this is caused mainly by *C. parvum*, a zoonotic species that has been reported to be “recently” adapted from a bovine to a human host environment, and *C. hominis*, formerly *C. parvum* genotype 1, and the species *C. canis*, *C. felis*, *C. meleagridis* and *C. muris* have been shown to infect other vertebrates [127,128]. The thick-walled oocyst is the stage responsible for transmission via fecal–oral or respiratory routes. The ingestion of this stage in contaminated water or food is associated with watery diarrhea, while its inhalation may provoke multiple symptoms in the upper and lower respiratory tract [129]. Remarkably, this protozoan infects the small intestine and develops into multiple stages at extracellular (sporulated oocysts-excysted sporozoites) and intracellular/extracytoplasmic (trophozoite-meront I-merozoite-meront II-merozoite-gamont-zygote) levels. Thus, within the *Cryptosporidium* life cycle, there are three critical steps: excystment, cell invasion and intracellular replication, which compromise intestinal homeostasis. 

The genome of *C. parvum* (Iowa strain) contains at least 20 genes encoding clan CA (papain-like) CPs of which 3 cathepsin L-like and 2 cathepsin B-like members belonging to the C1 family are referred to as “Cryptopains” [130]; however, only a few of them have been biochemically characterized. In this context, *C. parvum* encodes an otubain-like CP whose expression peaks in oocysts, decreases in sporozoites and differs in amino acid sequence from mammalian homologs. This has been identified and expressed as a recombinant enzyme [131]. Otubains or ubiquitin thioesterases are predicted CPs that cleave ubiquitin from Lys48- or Lys63-branched polyubiquitin chains serving multiple functions including protein recycling via the 20S proteasome and regulation of multiple processes involved in parasite survival. Cryptosporidial otubain-like is a 399 amino acid long enzyme that does not have a signal peptide or transmembrane domain (i.e., neither secreted nor cell surface anchored) with typical moieties such as the otubain-modified domain, the LxxL motif and the catalytic triad Asp19-Cys22-His173 along with an unusual C-terminal extension of 217 amino acids that is needed for enzyme activity. It is inhibited by ubiquitin aldehyde, N-ethylmaleimide and iodoacetic acid but intriguingly not by E-64 [131]. In other protozoan parasites, only a cytoplasmic otubain-like protease was described in *Leishmania infantum* with a preference for Lys48-linked substrate [132]. In the excystment process, proteolytic activities of the serine and cysteine-type proteases were detected in parasite homogenates from oocysts induced to excyst as assessed by the use of protease inhibitors such as PMSF and E-64; nevertheless, E-64 was unable to block excystation as was PMSF, ruling out a direct or functional involvement of CPs in this process [133].

Regarding cell invasion, *C. parvum* sporozoites were reported to harbor a 24 kDa protein with CP activity dependent on metallo-activation on the cell surface. This was suggested since some inhibitors including phosphoramidon, iodoacetic acid, EDTA and E-64 had an effect on its caseinolytic activity [134], indicating that it belongs to clan CA, C2 family of calpain-like proteases that depends on Calcium for activation. This molecule shares pH and metallo-dependent properties with host cell invasion by *Cryptosporidium*; nonetheless, further molecular studies aimed at its identification and molecular/functional characterization are still needed. More recently, another sporozoite-specific CP, a cathepsin L-like enzyme named “Cryptopain-1”, was cloned and expressed as a recombinant protein [135]. This protease is 401 amino acids long and lacks a typical signal peptide but possesses a preprodomain (165 amino acids long) including an intracytoplasmic domain and a transmembrane domain followed by a cathepsin propeptide inhibitor domain. This latter is not required for active enzyme folding in mature form (236 amino acids long), possibly by the presence of a 9 amino acid extension at the N-termini of the activated enzyme as occurs in *Plasmodium* falcipain-2 [136]. Cryptopain-1 is effectively inhibited by E-64 and was shown to degrade extracellular matrix components such as collagen and fibronectin, suggesting its role in parasite traffic into and egression from intestinal epithelial cells.

**Figure 3:**
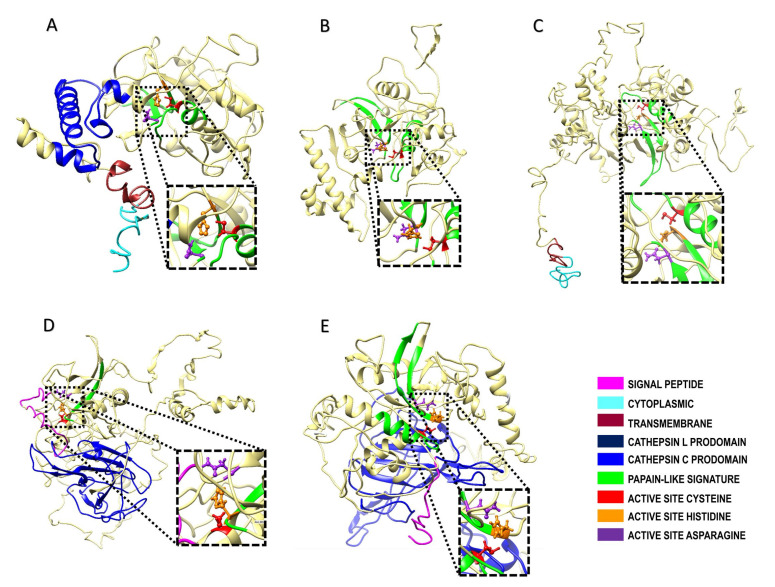
Protein structure models representing the Cryptopain family. The protein models of Cryptopain-1 (**A**), -2 (**B**), -3 (**C**), -4 (**D**) and -5 (**E**) were obtained using the I-Tasser server (https://zhanggroup.org/I-TASSER/; accessed dates: 28 February 2023, 3 March 2023, 22 and 28 July 2023), and the domains were identified with the InterPro platform (https://www.ebi.ac.uk/interpro/; accessed dates: 28 February 2023, 3 March 2023, 22 and 28 July 2023) and are indicated by colors at lower right. The catalytic triad Cys-His-Asn is displayed in ball-and-stick conformation and is magnified within dotted squares. Cryptopains 1-3 are cathepsin L-type and Cryptopains 4 and 5 are cathepsin B-type. From these analyses, Cryptopains 1 and 3 are predicted to be membrane-anchored (possess transmembrane domain), Cryptopain-2 is cytoplasmic (signal peptide and transmembrane domains absent) and Cryptopains 4 and 5 are secreted (possess signal peptide). Protein models were visualized and edited using the UCSF Chimera server v1.10.17.

In this review, we propose a complete denomination for members of the Cryptopain family (i.e., Cryptopain-1, -1a, -2, -3, -4 and -5) based on its cathepsin L-like (-1, -2 and -3) or cathepsin C-like (-4 and -5) type and following the nomenclature partially proposed by Ndao et al. [137] (Figure 3). Based on this, Cryptopain-1 corresponds to CryptoDB ID: orf cgd6_4880, which is derived from *C. parvum* strain Iowa II with sequence identity of 86.5% and 96.25% homology as compared with “Cryptopain-1a” (cubi_02618), which is derived from *C. ubiquitum* and displays identical positions of catalytic residues as well as transmembrane and cathepsin inhibitor domains. “Cryptopain-2” (CryptoDB ID: cgd3_680) is a 391 amino acids long enzyme containing three papain-like signature domains, one with an inferred cysteine active site and the two others with a likely active residue of histidine or asparagine (Figure 3). “Cryptopain-3” (CryptoDB ID: cgd7_2850) is a larger cysteine-type protease (673 amino acids long) containing a predicted transmembrane domain along with three papain-like signature domains; the positions of active site residues may be inferred from protein modeling analysis, while other specific domains are not obvious. “Cryptopain-4” (CryptoDB ID: cgd2_3320) is predicted to be an 816 amino acids long enzyme that contains a signal peptide (i.e., it is secreted) as well as a cathepsin C exclusion domain. From the catalytic triad, there is a typical active histidine inferred by domain analysis (Figure 3). Otherwise, “Cryptopain-5” (CryptoDB ID: cgd4_2110) is also significantly larger than Cryptopain-1 (635 amino acids long) and possesses an asparagine in the active site, while cysteine and histidine have been inferred in silico. This enzyme lacks a transmembrane domain but exhibits a signal peptide motif such as Cryptopain-4, suggesting that it is also a secreted CP; also, it exhibits a cathepsin C exclusion domain that confers possible exopeptidase activity. These data suggest that Cryptopains constitute a multifunctional family in this parasite, playing a key role(s) in the parasite life cycle and pathogenicity, although their expression might be stage-specific because oocysts express Cryptopain-1 and Cryptopain-2 but not Cryptopain-3 [137]; nevertheless, these issues should be solved in future studies.

As far as the regulation of *Cryptosporidium* CPs is concerned, some reports highlight at least one endogenous inhibitor and one leader compound that targets it. Indeed, the presence of endogenous CP inhibitors is physiologically critical to regulating the activities of endogenous proteases and to protecting the parasite from deleterious extracellular proteases [138]. Among these are the Chagasin-like inhibitors, a group that is different from typical mammalian inhibitors such as cystatins or thyropins but includes the protozoal orthologues of Chagasin, a small protein that tightly binds and regulates the endogenous activity of cruzipain (cruzain), a CP involved in differentiation and cell invasion by *Trypanosoma cruzi* [139]. Cryptostatin is the Chagasin ortholog in *C. parvum* (CryptoDB ID: cgd6_1910) that differs in sequence from other Chagasin-like members but shares three conserved motifs (NPTTG, GXGG and RPW/F). As a recombinant protein, cryptostatin was shown to be thermostable and able to bind and inhibit in the picomolar range papain, cathepsins B/L and Cryptopain-1. Interestingly, cryptostatin was found in the cytoplasm of oocysts and meronts but not in trophozoites, suggesting its involvement in host cell invasion but not during intracellular differentiation [22].

On the other hand, the vinyl sulfone compound K11777 (N-methyl-piperazine-Phe-homoPhe-vinylsulfone-phenyl; syn. APC 3316, K 777) is an irreversible and potent cathepsin B/L/S—but not cathepsin C—inhibitor with proven efficacy in experimental infections by protozoal and helminthic pathogens [140,141]. Likewise, the cryptosporicidal effect of this compound was evaluated at various levels [137]. The K11777 compound effectively inhibits *C. parvum* growth in MDCK cells; also, oocysts were killed in vivo after exposure to K11777. Further, in a model of immunocompromised mice highly susceptible to the infection with this parasite (IFNγ-receptor-KO), oral or intraperitoneal treatment with 210 mg K11777/kg per day prevents the lethal infection in these animals, which exhibited minimal intestinal inflammation without epithelial damage and remained parasite-free for 3 weeks; finally, the K11777 compound binds in silico and in vitro to the active site of Cryptopain-1 as expected from its activity-based mechanism of inhibition as it has been observed with other orthologous enzymes such as cruzipain and falcipains [137,142,143].

The lack of molecular and biochemical characterization of extracellular CPs in *Cryptosporidium* deserves further investigation, since at least two Cryptopains (-4 and -5, cathepsin B-type) are predicted to be secreted based on results of in silico analyses (Figure 3D,E).

## 5. *Blastocystis* Cysteine Proteases: From Subtypes to Pathogenic Mechanisms

The genus *Blastocystis* gathers heterokont (stramenopile) parasites including several species that cause gastrointestinal infection in humans and other hosts such as livestock, reptiles, amphibians, fishes, rodents, birds and insects. Due to its limited host specificity, all species causing human infection were formerly known as *B. hominis*. However, the current consensual terminology uses “*Blastocystis* sp. subtype nn (STnn)” to identify the distinct lineages, based on SSRNA sequence, causing infections in susceptible hosts [144]. In more recent reports, 22 subtypes have been identified from animal and environmental sources [145] where ST1-ST9 may infect humans and other hosts, while ST10-ST17, at least, is found in non-human species [146]. In the 3rd *International Blastocystis* Conference, 26 STs were reported (IBC, 2021) and by December 2022, up to 38 STs were identified [147]. It has been estimated that more than 90% of human infections are associated with ST1-ST4, ST3 being the most prevalent subtype [148,149]. This protozoan is transmitted through the fecal–oral route by the environmental cyst, which has a thick multilayered wall, and other stages alternate within the host’s large intestine (ameboid–vacuolar–multivacuolar and avacuolar–granular). Of these, the vacuolar form that is seen in cultures replicates by binary fission—though its genome reveals a nearly complete complement of meiotic genes—and precedes the cyst intermediate form, which is further shed and develops into an infective cyst. The ameboid stage, on the other hand, is considered the most virulent form of this parasite [150].

Although the pathogenicity induced by this parasite is still controversial due to the presence of asymptomatic individuals, there is an emerging consensus that *Blastocystis* sp. colonizing the host intestines may cause diarrhea, nausea, abdominal pain and even irritable bowel syndrome (IBS) [151]. An important factor related to these multiple symptoms is the enormous genetic variability seen among *Blastocystis* strains/subtypes. In this context, a comparative analysis based on the whole genome sequence of strains from ST1 (NandII strain, symptomatic human), ST4 (laboratory rodent) and ST7 (symptomatic human) showed substantial differences in genome size, GC content and protein-coding gene complement (ST1 > ST7 > ST4) as well as in mean genome size, intron-containing genes and mean exons per gene in the following order ST1 > ST4 > ST7 [152]. Likewise, the mean orthologue sequence identity ranged from 59% to 61%, supporting the fact that these STs are distinct species. Concurrently the number of secreted proteins is quite different, e.g., it is 89 in ST1 and 307 in ST7 [152,153]. Interestingly, the widely variant protein kinase and CP repertoires found among *Blastocystis* STs have been proposed as possible keys to explain the different virulence of isolates. [154].

The CP complement (80–100 genes per ST genome) not only is the most abundant protease type in this parasite but also displays great dissimilarity among *Blastocystis* STs (e.g., 39% of total proteases in ST7 and 47% in ST4) [155]. The families C1 (papain-like), C13 (caspase 1-like) and C19 (deubiquitinase-like) are extensively expanded genes; nevertheless, if C1 is common among protists, the number of C13 and C19 genes (11–16) found in *Blastocystis* is elevated as compared to other protists [156]. However, the implications of the differences found among STs at C56 (gamma glutamyl-type), C95 (amidophosphoribosyltransferase-like) and C97 (DeSI1 peptidase-like) families certainly deserve future studies.

In the last few decades, a number of studies have provided insights into the role of CPs during host–*Blastocystis* interactions. In rat-derived intestinal epithelial cell monolayers (IEC-6), *Blastocystis* sp. ST4 has been able to induce alterations in intestinal permeability, changes in host cell cytoskeleton at the level of filamentous actin and contact-independent induction of apoptosis [157,158].

**Figure 4:**
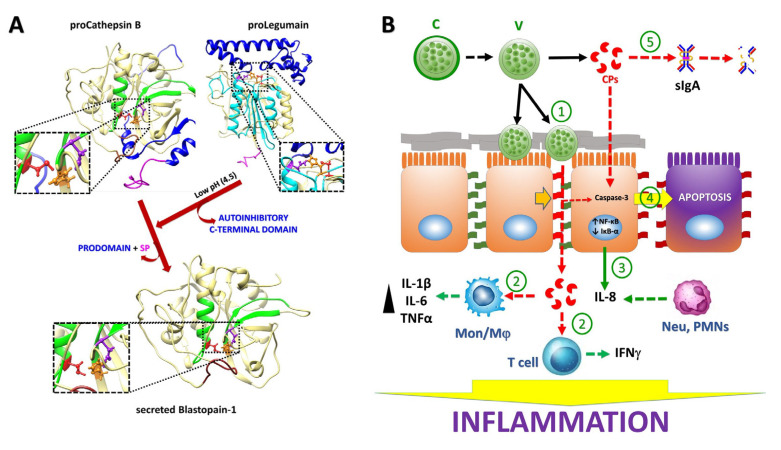
Blastopain-1 and other cysteine proteases from *Blastocystis*. Possible role in pathogenesis and immune evasion. (**A**) Protein structure models of zymogen and active forms of a secreted, Cathepsin B (Blastopain-1) and legumain from *Blastocystis* sp. The catalytic triad Cys(red)-His(orange)-Asn (purple) is displayed in *ball-and-stick* conformation and is magnified within the dotted squares Protein domains were obtained from the InterPro platform and are colored as follows: signal peptides (SP) in magenta; papain-like signature (CatB) in green; cathepsin C prodomain (CatB) and auto-inhibitory C-terminal prodomain (legumain) in blue; hemoglobinase C13 signature (legumain) in cyan; and occluding loop (CatB) in brown. Models were obtained using the I-Tasser server from sequences with a.n. CBK25506-2 (CatB) and CBK21815-2 (legumain). (**B**) Proposed roles of cysteine proteases from *Blastocystis*. The cyst (C) form precedes the vegetative vacuolar (V) form that alternates with other entities (ameboid, granular) at intestinal lumen where cysteine proteases (CPs) may be secreted and causes effects at different levels: (1) parasites attached at intercellular junctions may release CPs that degrade junctional proteins such as ZO1- and claudins, promoting increased epithelial permeability; (2) CPs at intraepithelial compartment might induce upregulation of proinflammatory cytokines in Monocytes/Macrophages (Mϕ) and T lymphocytes; (3) epithelial cells exposed to CPs produce IL-8, a potent chemoattractant for Neutrophils and Polymorphonuclear cells; and (4) disruption of intercellular junctions along to a likely activation of Caspase-3 pathway may result in programmed cell death (apoptosis). Also, secreted CPs are able to degrade secretory IgA in vitro (5), an important effector in the mucosal system that has been observed at increased levels in symptomatic cases of Blastocystosis.

The damage to epithelial barrier function by *Blastocystis* sp. may vary, at least in vitro, based on the ST´s virulence and is possibly linked to host specificity. In this context, the use of human colorectal adenocarcinoma cells (CaCo-2) showed that ST4 (from rodents) was not able to increase permeability in this cell line as it did in IEC-6 cells. On the other hand, ST7 (from humans) effectively provoked increased monolayer permeability (i.e., altered ZO-1 integrity/distribution) and apoptosis in CaCo-2 cells as assessed by phosphatidylserine externalization, nuclear fragmentation and increases in expression of Caspase-3 and Caspase-9 but not Caspase-8, thus involving a mitochondrial (intrinsic) mechanism of programmed cell death [159]. In addition, *Blastocystis* ST7 was shown to adhere to intercellular spaces, causing a disruption of tight junction proteins such as ZO-1 and claudins. Interestingly, metronidazole-resistant isolates of this ST with lower adhesion ability provoked fewer effects in the epithelial cells [160]. In the genome sequence of *Blastocystis* ST7, 29 CPs are predicted to be secreted (16 C1, 11 C13, 1 C39 and 1 C45 family members); of these, 1 C1-type cathepsin B-like (acc. number: CBK25506-2; 321 amino acids long, MW 35.6 kDa, proenzyme, and 27.9 kDa, secreted; and herein named “Blastopain-1”) and a C13-type legumain (hemoglobinase family, a.n.: CBK21815-2) were detected in the medium in which parasites were cultured [161]. Interestingly, the recombinant legumain activated the recombinant Blastopain-1, which was then able to induce a significant increase in permeability to FITC-labeled dextran across CaCo-2 cell monolayers, similar to the effect displayed by the parasite culture supernatants, and the E-64 inhibitor abolished this effect [162]. These observations suggest an active role of CPs in the disruption of epithelial intercellular junctions and eventually in apoptotic induction by *Blastocystis* (Figure 4A,B).

Regarding immunologic mechanisms, experimental infections in rats with cysts from the rodent strain RN94-9 (ST4) resulted in chronic infection at cecal mucosa with mild goblet cell hyperplasia (neutral mucin^+^ phenotype) and upregulation of type-1 response mediators such as IFNγ and proinflammatory cytokines such as IL-12 and TNFα, showing the potential of this parasite to elicit inflammatory as well as specific local responses involving T Cells and monocytes/macrophages [163]. In macrophages of murine origin, serine proteases from *Blastocystis* ST7 induced the upregulation of IL-1β, IL-6 and TNF-α in a MAP kinase-dependent signaling cascade (ERK/JNK), while CPs induced a similar response in a MAPK-independent pathway [164]. Accordingly, human colonic epithelial HT84 cells exposed to CPs from *B. ratti* WR1 displayed Interleukin-8 upregulation through the regulatory protein complex NFκB with concomitant degradation of its antagonistic complex IκB-α [157]. Also, cleavage of human secretory IgA in vitro by *Blastocystis* isolates B (human) and WR1 (rodent) was significantly sensitive (>50%) not only to CP inhibitors such as iodoacetamide but also to aspartyl protease inhibitors (pepstatin A) [158].

Recent studies open the proposal that CPs might be secreted by *Blastocystis* sp. within extracellular vesicles as it occurs in other intestinal protozoa such as *G. duodenalis* [31] and *E. histolytica* [165]. *Blastocystis* STs 1–3 secrete exosomes (30–100 nm sized) that, when added to human leukemia monocytic cells (THP-1 line), caused different effects on the expression of proinflammatory (IL-6 and TNF-α) and anti-inflammatory (IL-4 and IL-10) cytokines: ST1 upregulated IL-6 and TNF-α concomitant to IL-4 and IL-10 downregulation, ST2/ST3 downregulated IL-10 and ST3 upregulated IL-6 [166]. Taken together, all of these data provide insights into the important roles of *Blastocystis*´ CPs contributing to immunopathological responses such as urticaria and bowel inflammation observed in some patients [167] and leading to immune evasion and modulation that promotes colonization and persistence of *Blastocystis* in susceptible hosts (Figure 4B). 

The notion that CPs expressed and secreted by *Blastocystis* are promising candidates as markers of pathogenicity and therapeutic targets is tempting based on experimental evidence. However, associations of differential protease activities of a given ST with clinical outcomes in blastocystosis cases have been reported but are inconclusive by the biological plasticity of the parasite. In previous zymography analyses, the activity of 32 kDa protease(s) was detected preferentially in isolates from symptomatic individuals as compared with those from asymptomatic ones, all belonging to the ST3 [168]. Recent reports testing the ST1–3 and ST6 isolates showed that protease activity was also higher in isolates from symptomatic individuals as compared to asymptomatic carriers, with ST6 displaying the highest activity and ST2 the lowest; however, there was no relation with the induction of proinflammatory markers such as IFNγ, IL-6, IL-8, IL-12, TGFβ and TNF-α in HT-29 cells [169]. Recently, zymography analyses of *Blastocystis* sp. sampled from water (ST1–ST4) and animals including pigs (ST5), ducks and chickens (ST7) revealed that CPs were the most abundant protease type and were related to animal samples, but interestingly, the zymography patterns may change over time [170]. In this context, differences in CP activities have been observed between STs, e.g., avian ST7 isolates, which showed an activity twice higher than the rodent ST4 ones, along with variations in protease activities in cultures from a single isolate [154]. Likewise, comparisons of the haplotypes of a region (amino acids 69–219) in relation to the secreted cathepsin B (Blastopain-1) between asymptomatic carriers and IBS cases allowed the classification of isolates into clades in which the isolates from clinical cases displayed higher polymorphisms; nonetheless, a relation with pathogenicity was not inferred [171].

Collectively, these observations suggest that the host–parasite interplay during blastocystosis might modulate the production/secretion of parasitic virulence factors, such as CPs, although host-related factors, including the innate intestinal microenvironment, immune responses and even “intercellular communication” between host and parasite (via EVs as exosomes) should not be excluded.

## 6. Conclusions and Future Directions

The diseases caused by *Giardia* (giardiasis), *Entamoeba* (amoebiasis), *Cryptosporidium* (cryptosporidiosis) and *Blastocystis* (blastocystosis) represent a major burden in humans worldwide. Molecular variation within and among these protozoans plays a key role in host–pathogen interactions. Virulence factors are of particular importance and include secreted CPs, which have a wide range of substrates and can directly affect and destroy tissues and cells to enable parasite establishment and/or invasion. 

This review shows that CPs, particularly members of the CA superfamily, are important virulence factors. These include members of classes C1 (cathepsins B/L-type) and C2 (calpain-like proteases), which are involved in pathogenesis and apoptosis-like induction, respectively. Moreover, most cathepsin L-like proteases relate to stage-specific localization and/or host invasion (e.g., Cryptopains 1–3), while important cathepsin B-like proteases (e.g., Cryptopains 4–5, Blastopain-1, Giardipain-1 and EhCPs 1–2, 4–5 and EhCP112) possess signal peptides and are secreted. Interestingly, cathepsin L-like proteases only have endopeptidase activity, whereas cathepsin B-like proteases, harboring an additional occluding loop, both have carboxy(exo)peptidase (at low pH) and endopeptidase (at high pH) activities; therefore, the versatility of cathepsin B-like activities correlates with extracellular localization, where interactions with more potential substrates occur as compared with surface-bound or intracellular CPs. In this context, CPs with similar features and secreted by different intestinal protozoa promote tissue degradation processes and invasion of intestinal cells, as observed for other protozoan parasites [12].

The pathway by which secreted virulence factors are exported and delivered to sites of action requires further studies. Emerging evidence suggests that even molecules lacking signal peptides are secreted in EVs, particularly via exosomes. EVs might promote parasite establishment and modulate immune responses; CPs have been identified as cargos of EVs of *Blastocystis*, *Entamoeba* and *Giardia* but not *Cryptosporidium* [31,165,166]. This is now a promising field for future work. 

Although recognized as key factors, the relationship between CPs secreted by these parasites and disease requires further investigation. In this context, higher CP activities have been found in invasive isolates versus non-invasive isolates from individuals with amoebiasis [117]. Also, higher protease activities have been reported in isolates recovered from symptomatic individuals with blastocystosis as compared to asymptomatic ones [169], while protease repertoires differ between *Giardia* isolates from symptomatic and asymptomatic patients [172]. These observations reveal the importance of parasite “plasticity” and host factors, particularly innate and adaptive immune responses, to modulate or control the virulence of these pathogens (reviewed in [4]).

**Figure 5:**
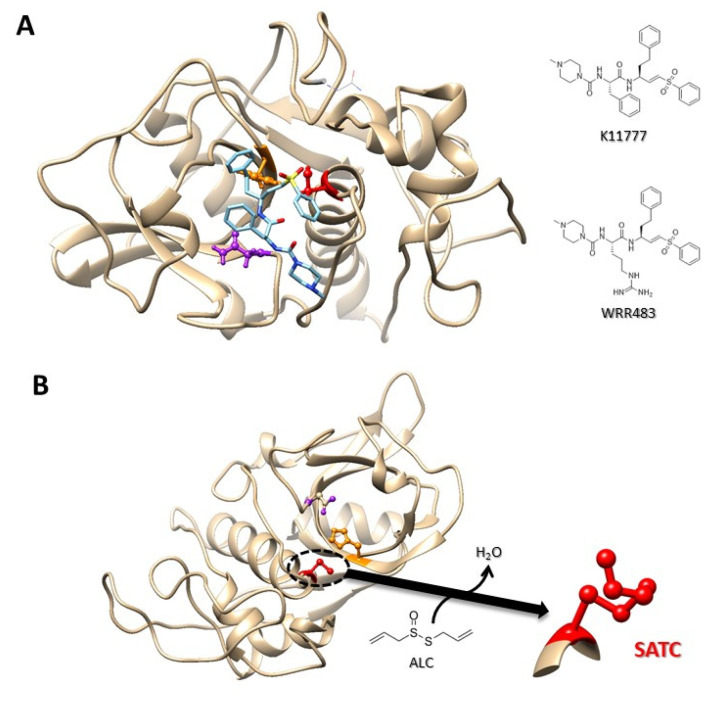
Mechanisms of inhibition of CPs from intestinal protozoa by synthetic and natural compounds. Protein models of secreted CPs were obtained by homology modeling using the Phyre2 server. (**A**) Blockade of the active site of EhCP1 by the vinyl sulfone K11777. Prediction model from SwissDock server (http://www.swissdock.ch/docking; accessed dates: 28 March 2023 and 11 July 2023) of a favored docking position (ΔG = −7.2436kCal/mol) of K11777 (in cyan, displayed in *stick* conformation) at the vicinity of the catalytic triad of EhCP1 displayed in ball-and-stick conformation (cysteine in red, histidine in orange and asparagine in purple). K11777 structure was obtained in SMILES format with further energy minimization using Avogadro suite v1.2. As reference, chemical structures of vinyl sulfone inhibitors K11777 and WRR483 are shown on the right. (**B**) Modification of active site cysteine from Giardipain-1 by allicin. The protein backbone shows the positions of catalytic residues that are displayed as described above. Upon interaction with allicin (ALC), the catalytic cysteine is converted into S-allylthiocysteine (SATC), which lacks nucleophilic nature as the cysteine thiol, thereby inactivating Giardipain-1. In this case, other thiol-disulfide exchange reactions could proceed with allosteric cysteines, perturbing enzyme activity. Protein models were visualized and edited using the UCSF Chimera server v1.10.17.

The presence of endogenous regulators of CP activity (mainly cystatins) in these protozoa [22,24] (protein ID: ACA1_096350 in *E. histolytica*) is critical to avoid unwanted intracellular proteolysis, but at the extracellular level, exogenous inhibitors are needed to protect the host. To date, the vinyl sulfone inhibitors K11777 and WRR483, the former targeting Cryptopain-1 and both inhibiting EhCP1 by binding to the active cysteine site [75,137] (Figure 5A) as well as the garlic derivative allicin (diallyl thiosulfinate), which inhibits Giardipain-1 activity likely via a thiol–disulfide exchange mechanism involving active site cysteine [173] (Figure 5B), constitute a growing repertoire of inhibitors against protozoan CPs, emphasizing the need for further discovery. This work could benefit from advances obtained for other pathogens [174].

## Figures and Tables

**Table 1:** Characteristics of *Entamoeba histolytica* cysteine proteases (EhCPs).

Cysteine Proteinase	Celular Localization	Involved in …	References
EhCP1	Cytoplasmic large vesicles different to those of EhCP3	Adhesion and cytopathic effect Proteolysis of collagen type-1, villin, IgA, IgG, C3, pro-IL-18 Degrades erythrocytes and hemoglobin	Scholze and Schulte, 1988 [61]; Li et al., (1995) [73]; Zhang et al., (2000) [74]; Melendez-López et al., 2007 [75]; Irmer et al., (2009) [76]
EhCP2	Membrane Move to phagocytic vesicles during erythrophagocytosis	Adhesion and cytopathic effect Proteolysis of collagen, proteoglycan, pro-IL-1β, chemokines CXC and CCL Degrades erythrocytes and hemoglobin	Li et al., (1995) [73]; Zhang et al., (2000) [74]; Irmer et al., (2009) [76]; Que et al., (2002) [77]; Pertuz-Belloso et al., (2004) [78]
EhCP3	Cytoplasmic large vesicles different to those of EhCP1. Move to phagocytic vesicles during erythrophagocytosis	Probably in digestion of nutrients	Que et al., (2002) [77]; Serrano-Luna et al., (2013) [79]; Gastelum-Martínez et al., (2018) [80]
EhCP4	Nucleus, perinuclear endoplasmic reticulum and cytoplasmic acidic compartment	Adhesion and cytopathic effect Proteolysis of laminin, villin, IgA, C3, Pro-IL-1β Pathogenesis of intestinal invasive amoebiasis	Li et al., (1995) [73]; Zhang et al., (2000) [74]; He et al., (2010) [81]
EhCP5	Membrane	Proteolysis of collagen, fibrinogen, mucin, pro and mature IL-18 Degrades erythrocytes and hemoglobin Secretagoge activity Pathogenesis of invasive amoebiasis	Jacobs et al., (1998) [63]; Que et al., (2003) [77]; Hellberg et al., (2002) [82]; Moncada et al., (2006) [83]; Thíbeaux et al., (2012) [84]; Cornick et al., (2016) [85]
EhCP6		Stress reponse	Park et al., (2001) [86]; Ghosh and Raha (2015) [87]
EhCP112	Membrane, forming a complex with EhAdh112	Degrades erythrocytes and hemoglobin Proteolysis of claudin 1 and claudin 2 at the tight junctions Proteolysis of β-cat, E-cad, Dsp l/ll, and Dsg-2 in adhesion junctions and desmosomes	Irmer et al., (2009) [76]; Ocádiz et al., (2005) [88]; Cuellar et al., (2017) [89]; Hernández-Nava et al., (2017) [90]

## Data Availability

Data sharing not applicable. No new data were created or analyzed in this study. Data sharing is not applicable to this article.
